# The Canadian contribution to the otolaryngology literature: a five year bibliometric analysis

**DOI:** 10.1186/s40463-014-0047-1

**Published:** 2014-11-22

**Authors:** Joshua Gurberg, June RJ Lin, Elaheh Akbari, Paul White, Desmond A Nunez

**Affiliations:** Division of Otolaryngology, Diamond Health Care Centre, The University of British Columbia, 4th Floor, 2775 Laurel Street, Vancouver, BC Canada V5Z 1 M9; Department of Engineering Design and Mathematics, The University of the West of England, Bristol, UK

**Keywords:** Bibliometry, Otolaryngology, Research, Productivity, Nationality, Canadian, Canada

## Abstract

**Objectives:**

To assess the 2008–2012 Canadian contribution to the Otolaryngology literature.

**Methods:**

All articles published from January 2008 - December 2012 in 5 Otolaryngology journals were reviewed. Nationality, number of authors, and study type were extracted. The output, number of authors, and study type of Canadian papers were compared to International papers using Mantel-Haenszel Common Odds Ratio Estimate, Pearson’s Chi-Squared or Fishers exact tests.

**Results:**

4519 papers were analyzed. There was a statistically significant decrease in Canadian authored papers from 12.8% in 2008–9 to 10.2% in 2011–12 (Fishers exact, p = .01). Multi-authorship increased in Canadian papers (χ2, p = .01). The types of studies published by Canadian Otolaryngologists did not change over the study period.

**Conclusions:**

Canadian authored papers in a sample of Otolaryngology journals decreased from 2008 to 2012. The increase in multiauthorship, whilst indicating increasing collaboration, suggests reduced per capita publication productivity. These findings warrant further study.

## Background

The future of Otolaryngology – Head & Neck surgery relies on ongoing knowledge creation. Bibliometry, defined as the quantitative evaluation of scientific literature, is an accessible tool to evaluate the progress of the specialty [1]. Several studies have evaluated scientific output trends in Otolaryngology; however, there are limited studies specifically quantifying the Canadian contribution to the Otolaryngology literature [1-5]. In 2012 there were 210 post-MD trainees, 74 full-time faculty, and 198 part-time faculty in the thirteen University based academic Otolaryngology programs distributed across Canada [6,7]. The primary funding source for medical research in Canada is the Canadian Institutes of Health Research (CIHR), and funds are allocated on a competitive basis. Biomedical and health care research revenues of Canadian faculties of Medicine amounted to 2 698 656 000 Canadian Dollars in 2009-10 [7]. The primary medical research funding bodies are the National Institutes of Health (NIH) in the United States (U.S.), and the Medical Research Council and National Institute for Health Research (NIHR) in the United Kingdom (U.K.), respectively. According to the Organization for Economic Cooperation and Development, in 2013 the gross domestic expenditure on research and development as a percentage of gross domestic product was 1.74% in Canada, 2.77% in the United States, and 1.77% in the United Kingdom. Unfortunately, it is not possible to ascertain the exact funds allocated to Otolaryngology research endeavors for each nation.

This study will assess the 2008–2012 Canadian contribution to the global Otolaryngology – Head & Neck Surgery literature, evaluating the types of articles published and number of authors per paper.

## Methods

**Figure 1:**
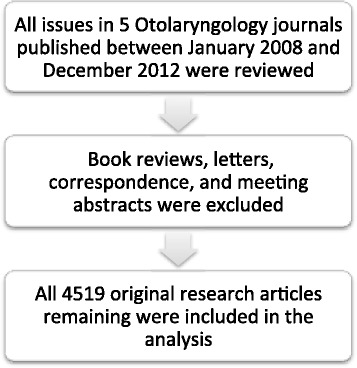
**Article acquisition.**

This study was exempted from institutional review board ethics approval, as no patient data was included. The full manuscripts of all original articles published by 5 leading Otolaryngology journals from January 2008 to December 2012 were reviewed (see Figure 1). These were chosen based on their most recent impact factor (IF) ranking in 2013, favoring general Otolaryngology journals. The included journals were: (1) The International Journal of Pediatric Otorhinolaryngology; (2) Head & Neck; (3) The Journal of Otolaryngology – Head and Neck Surgery; (4) Archives of Otolaryngology; and (5) Clinical Otolaryngology. The Journal of Otolaryngology – Head and Neck Surgery was selected because, as the only Canadian Otolaryngology journal, it presumably captures a large proportion of Canadian research output.

The data was extracted independently by four authors (JG, RJL, EA and DAN). The table of contents of each journal issue was reviewed and book reviews, letters, correspondence, and meeting abstracts were excluded, leaving only original articles, all of which were included in the study (see Figure 1). Data recorded from each article included: number of authors, types of research, year of publication, country from which the work originated, name of the Canadian department from which the work originated, name of the Canadian author, and major funding resources if indicated in the article. A Canadian article was defined as any article submitted from a department with a Canadian mailing address, any article in which the corresponding author was from a Canadian department, or any article involving international collaboration where at least one author had a Canadian mailing address. Other designations included United States, United Kingdom, and other. The number of authors was grouped as 1, 2, 3 to 4, and over 5.

Studies were classified as randomized control trials, other clinical studies, case reports, primary basic science research, secondary research, or other. Other clinical studies included prospective studies, retrospective reviews, and case series of 3 patients or more. Primary basic science research included in vivo, in vitro, and cadaver studies. Secondary research included systematic and narrative review articles as well as meta-analyses. All articles not fitting the aforementioned criteria were considered “others”.

The investigators undertook a period of training in article classification. In brief, concordance of the investigators’ article classification was initially checked by calculating Fleiss Kappa inter-rater reliability on a series of 50 journal articles, which each reviewer assessed independently. The articles that were discordantly classified were discussed by the investigators and a single classification agreed by consensus. A further 50 articles were then similarly classified and inter-rater Fleiss Kappa concordance calculated until the concordance level exceeded 0.8.

Statistical analysis was performed using the SPSS version 20 software package. The nationality of origin, number of authors per article, and study types of papers in 2008 and 2012 were compared using the Pearson chi-squared test or fischer exact test with a level of significance set at p < 0.05. These parameters were further analyzed using the common odds ratio estimate and by comparing years 2008 and 2009 to 2011 and 2012.

## Results

### Nationality

4519 articles meeting the inclusion criteria were published in the 5 journals over the period studied; 530 (11.7%) originated from Canada. There was a statistically significant decrease in Canadian authored papers from 12.8% in the combined years of 2008/09 to only 10.2% in 2011/12 (*χ*^2^, *p* < .05).

### Number of authors

**Figure 2:**
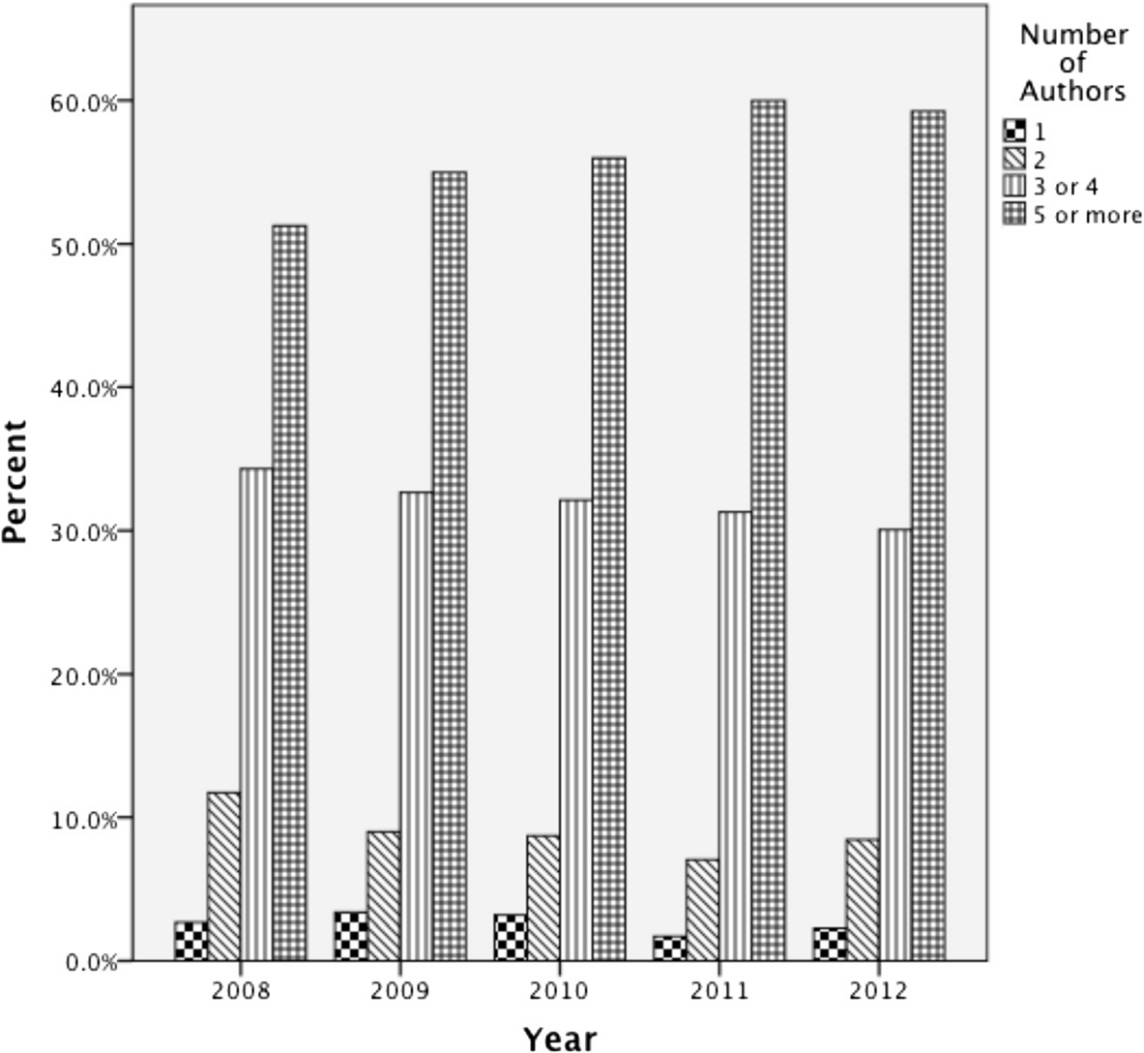
**Number of authors per article over time.**

Across all nationalities, there was a decrease in the proportion of dual authorship articles and an increase in articles with five or more authors (see Figure 2). In 2008, 51.3% of the sample journal articles were authored by 5 or more individuals. By 2012 this had increased to 59.6%, representing a significant change (*χ*^2^, *p* = .001). Articles in 2012 were approximately 1.4 times more likely to have 5 or more authors when compared to those published in 2008 (Odds ratio = 1.3, 95% CI 1.15 to 1.66). When comparing Canadian to non-Canadian authored papers in 2008, the percentage of Canadian authored papers with 3 or more authors (76.5%) was significantly lower than the percentage of non-Canadian papers with 3 or more authors (87.2%) (*χ*^2^, *p* < .05) (see Table 1). By 2012 the percentage of Canadian papers with 3 or more authors (90%) was comparable to that of non-Canadian papers (89.2%). There was a significant increase in multi-authorship in Canadian authored papers from 2008 to 2012 (*χ*^2^ [trend], *p* = .014).

**Table 1:** **Number of authors per article over time by nationality**

**Year**			**Authors**		**Total**
			**1 or 2**	**3 or more**	
2008	Not Canadian	Count	103	699	802
		Percentage	12.8%	87.2%	100.0%
	Canadian	Count	32	104	136
		Percentage	23.5%	76.5%	100.0%
2009	Not Canadian	Count	98	694	792
		Percentage	12.4%	87.6%	100.0%
	Canadian	Count	12	86	98
		Percentage	12.2%	87.8%	100.0%
2010	Not Canadian	Count	94	678	772
		Percentage	12.2%	87.8%	100.0%
	Canadian	Count	11	101	112
		Percentage	9.8%	90.2%	100.0%
2011	Not Canadian	Count	61	717	778
		Percentage	7.8%	92.2%	100.0%
	Canadian	Count	16	88	104
		Percentage	15.4%	84.6%	100.0%
2012	Not Canadian	Count	91	754	845
		Percentage	10.8%	89.2%	100.0%
	Canadian	Count	8	72	80
		Percentage	10.0%	90.0%	100.0%

### Study type

**Figure 3:**
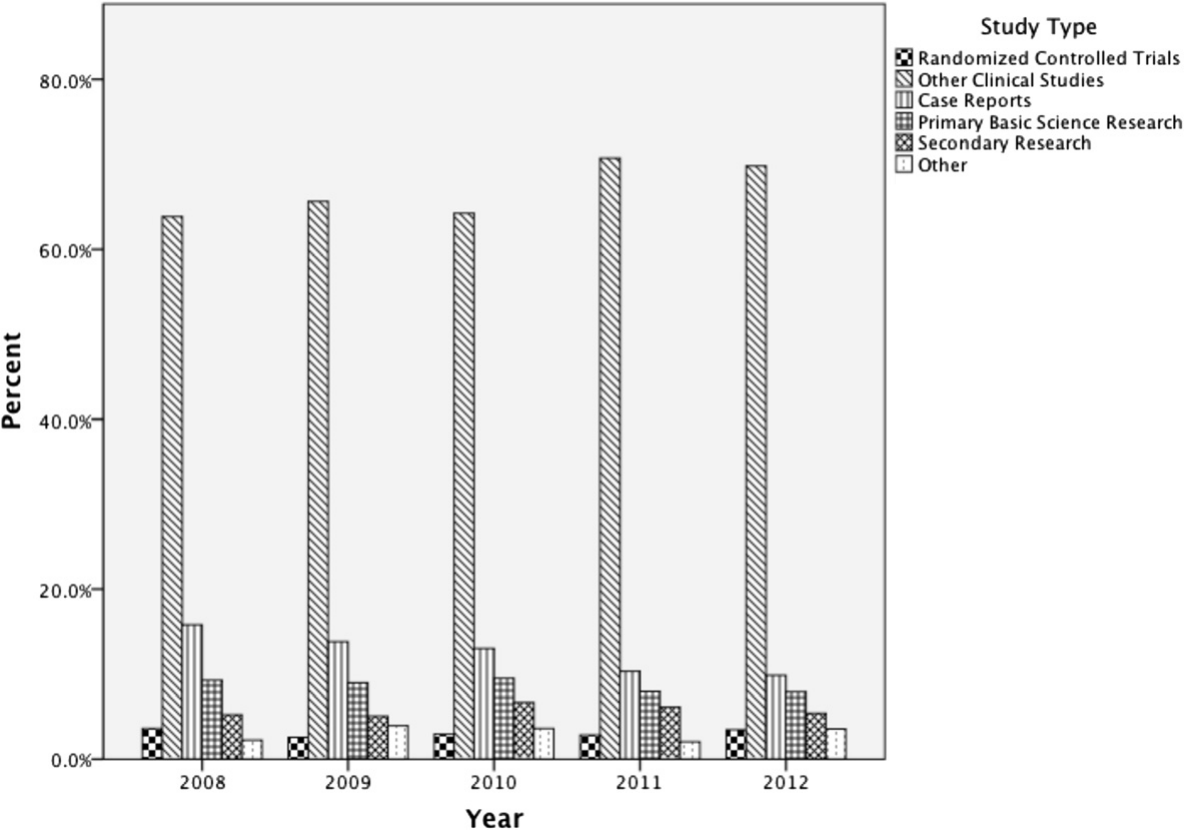
**Proportion of study types published over time.**

**Figure 4:**
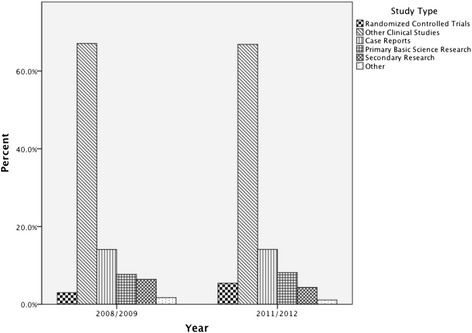
**Proportion of study types published by Canadian authors over time.**

The Inter-rater reliability amongst the four reviewing authors after training was high (Fleiss Kappa = 0.89). The percentage of case reports decreased over time (see Figure 3). Interestingly, the types of studies published by different nations is significantly different (p < 0.05) (see Table 2). Canadian and American authors publish a greater number of case reports and primary basic science research articles, while UK authors publish more secondary research. When the combined publications of 2008 and 2009 are compared to 2011 and 2012, there was no change in the study types published by Canadian authors (p = 0.759) (see Figure 4).

**Table 2:** **Proportion of study types by nationality**

		**Study rype**					
		**RCTs**	**Other clinical studies**	**Case reports**	**Primary basic science research**	**Secondary research**	**Other**
Nationality	Canadian	21	351	72	49	27	10
		4.0%	66.2%	13.6%	9.2%	5.1%	1.9%
	American	22	837	210	135	56	51
		1.7%	63.8%	16.0%	10.3%	4.3%	3.9%
	UK	12	198	23	5	37	47
		3.7%	61.5%	7.1%	1.6%	11.5%	14.6%
	Other	85	1635	263	205	137	31
		3.6%	69.4%	11.2%	8.7%	5.8%	1.3%

RCT = randomized controlled trial.

## Discussion

The results indicate that the Canadian contribution to the 5 Otolaryngology journals sampled diminished over time from 12.8% in 2008/09 to 10.2% in 2011/12. At the same time, there was a significant increase in Canadian authored papers with five or more authors, while the type of research being undertaken by Canadian Otolaryngologists did not change.

The authors recognize that this paper does not represent a comprehensive analysis of all Otolaryngology research output for Canada from 2008 to 2012 as meeting abstracts were not included, only a sample of primarily general Otolaryngology journals were reviewed, and many ENT researchers publish their work in non Otolaryngology, open-access, and other sub-specialty journals [1,4]. That being said, the journals chosen were amongst the most influential general journals for our specialty and included the Canadian Otolaryngology journal that should theoretically capture a large proportion of the output of Canadian Otolaryngologists. Whilst we did not review any journals with only an online presence, which are believed to be at the forefront of the open access movement, this shortcoming should not have preferentially affected the findings related to the research output of Canadian Otolaryngologists. All authors, regardless of national origin, would have the opportunity to publish in these journals and so the effect should not result in differential changes in national publication rates in the traditional journals. Our findings are therefore concerning, as taken together, the data suggests reduced per capita publication productivity by Canadian Otolaryngologists. This raises several important questions. Are the findings valid and will they be replicated in a wider journal survey? What accounts for this decrease in productivity? Are there feasible solutions?

In order to assess whether Otolaryngology research productivity is diminishing in general, a literature search for other bibliometric studies of Otolaryngology as well as other surgical specialties was undertaken. In 1999, Scarney and colleagues reviewed 10 leading Otolaryngology journals and found that the UK contribution was stable at 20% from 1985 to 1994 [4]. There was also a significant change toward the publication of clinical research rather than pure laboratory studies, as well as a significant increase in multi-authorship publications (3 or more authors) over the study period [4]. Similarly, Sandhu and Wright found no growth in the output of Otorhinolaryngological publications from January 1997 to December 1999 in the United Kingdom [3]. Decreasing research output, therefore, is not isolated to Canada. In 2005, Cimmino and colleagues reviewed 29 major Otolaryngology journals from 1995–2000. An analysis of papers/million population had Canada falling behind with 3.9 compared to 6.0 and 7.3 in the United States and United Kingdom respectively [1]. On the other hand, when adjusted for GDP, the three regions normalized to approximately 0.2 papers/GDP [1]. GDP is an important determinant of national research funding, and may account for the differences in publication output.

Funding is crucial to research production, as demonstrated by a study published in the Laryngoscope in 2012, which demonstrated that faculty members who received NIH funding had significantly greater research productivity and impact than non-funded authors [5]. The impact of cuts to research funding in the United Kingdom further supports this finding [2,4]. Increasing service demands have also been implicated in decreasing research output [2]. One interesting study looked at publication trends among Orthopedic Surgery residents in the United States before and after the institution of resident work-hour regulations and found that there was a greater probability of peer review authorship in every resident year after work-hour restriction than before [8].

## Conclusions

To the best of our knowledge, this study represents the most current comprehensive analysis of Canadian Otolaryngology research output. The finding suggesting decreasing research output for Canadian Otolaryngologists between 2008–2012 requires further study. One may speculate that many of the issues discussed above apply to Canada, including difficulty in obtaining highly competitive grant funding and heavy service demands for Canadian Otolaryngologists and trainees. Further research is needed to support these findings in a wider journal survey as well as to determine potential causes and solutions to ensure a strong future for academic Otolaryngology – Head & Neck surgery in Canada.
